# Anion Exchange Membranes for Fuel Cells Based on Quaternized Polystyrene-*b*-poly(ethylene-*co*-butylene)-*b*-polystyrene Triblock Copolymers with Spacer-Sidechain Design

**DOI:** 10.3390/polym14142860

**Published:** 2022-07-13

**Authors:** Qun-Gao Chen, Ming-Tsung Lee

**Affiliations:** Department of Chemical Engineering and Biotechnology, National Taipei University of Technology, Taipei 10608, Taiwan; t110738046@ntut.edu.tw

**Keywords:** anion exchange membrane, hydroxide ion transport, self-assembly, nano-segregation, ion conductivity, morphology, dissipative particle dynamics

## Abstract

This work studied the polystyrene-*b*-poly(ethylene-*co*-butylene)-*b*-polystyrene (SEBS) triblock copolymers functionalized by butyl quaternary ammonium (C_4_Q) groups and alkyl side chains of different chain lengths (C_n_, n = 0 to 24). The hydrated membrane morphology was modeled by dissipative particle dynamics simulation at hydration levels from 10 to 30. A hydroxide model was devised to characterize the diffusivity of anions under the coarse-grained framework. In general, the ionomers with alkyl side chains provided ion conductivity of a similar level at a lower ion exchange capacity. All hydrated SEBS–C_4_Q–C_n_ ionomers showed clear phase separation of the hydrophobic and hydrophilic domains, featuring 18.6 mS/cm to 36.8 mS/cm ion conductivity. The hydrophilic channels expanded as the water content increased, forming more effective ion conductive pathways. Introducing excess alkyl side chains enhanced the nano-segregation, leading to more ordered structures and longer correlation lengths of the aqueous phase. The membrane morphology was controlled by the length of alkyl side-chains as well as their tethering positions. Ionomers with functionalized side chains tethered on the same block resulted in well-connective water networks and higher conductivities. The detailed structural analysis provides synthesis guidelines to fabricate anion exchange membranes with improved performances.

## 1. Introduction

The zero-carbon-emission nature and its versatility have made fuel cell (FC) technology essential in developing affordable clean energy sources [1]. To manufacture low-cost fuel cell stacks, researchers study the anion exchange membrane (AEM) due to its alkaline working conditions allowing non-precious metals as electrocatalysts. However, commercializing AEMFC is currently hindered by issues in performance and durability compared with the more successful ones based on the proton exchange membrane (PEM). The diffusion coefficient of hydroxide ions is 1.7 times lower than that of protons [2]. Thus, a designed AEM should provide a more ion-conductive network than the inter-connective hydrophilic clusters of Nafion PEM [3] to deliver the same level of ion conductivity. Moreover, the performance of AEM decays due to the chemical degradation of ion-conducting parts from the attack of hydroxide ions [4,5,6]. Novel approaches to overcome these drawbacks are constantly reviewed, including synthesis strategies [7,8,9,10,11], ion conductivity [12], cell performance [11,13,14,15], and the stability of functional groups [16,17]. Research efforts have pushed the peak power density of AEMFC to approach that of the state-of-the-art PEMFC, exceeding 1.5 Watt/cm^2^ at 80 °C. However, the lifetime of AEMFC is commonly lower than 1000 h, while the performance drops significantly in the first 200 h [6,13].

The degradation happens in cationic groups [18] and the backbone [19] of polymer electrolytes that comprise AEM. For the cationic groups, the nucleophilic attack from hydroxide ions surrounds the electronegative atoms and therefore decreases the ion exchange capacity (IEC) of AEM [20,21]. Introducing alkyl chains as spacer provides steric hindrance and alters the degradation pathway [20,21]. For the polymer backbone, the attack occurs on electron-withdrawing groups such as sulfone linkages [22,23] and therefore causes the backbone cleavage and affects the mechanical properties of AEM. Synthesizing membranes based on aryl-ether-free materials improves durability. Consequentially, polystyrene-*b*-poly(ethylene-*co*-butylene)-*b*-polystyrene (SEBS) functionalized by quaternary ammonium (QA) [13] has become a popular choice in constructing alkaline-stable AEM, and its significance has drawn a great deal of attention in the past few years [20,24,25,26,27,28,29,30,31,32,33,34,35,36,37,38,39,40].

The backbone of SEBS consists of all C–C bonds, making it thermodynamically stable even in high alkaline conditions. Moreover, the immiscibility of the PS chain and the PE-PB chain of triblock SEBS drives the nano-scaled segregation even in the dry polymer state [28,41]. The ordered structure makes it convenient to create ion conductive materials after post-polymerized functionalization. Previously, Zheng et al. [39], Sun et al. [42], and Zhou et al. [43] fabricated quaternized SEBS (QSEBS) by chloromethylated SEBS, followed by a substitution using trimethylamine. In these studies, QSEBS with IEC of 0.3 mmol/g [39] and 0.66 mmol/g to 1.54 mmol/g [42,43] reached ion conductivity of 9.3 mS/cm at 80 °C [39] and 3.0 mS/cm to 9.6 mS/cm at 20 °C, respectively [42,43]. Water uptake (WU) increases proportionally with IEC and the swelling ratio. AFM phase images show well-defined nano-segregation of hydrated QSEBS, where PS blocks with pendent TMA groups form continuous ion conductive channels with water, and the channels expand when the membrane is swollen [42]. For QSEBS (30% PS block, IEC of 1.35 mmol/g) with 52 wt% WU, the size of ionic clusters varies from 57 nm to 95 nm [37]. Such morphology is related to efficient ion transport, which can be increased by elevating the degree of functionality. The hydroxide ion conductivity reached 100 mS/cm at 80 °C with IEC of 1.36 mmol/g in the study by Gao et al. [29], 180 mS/cm at 70 °C with IEC of 1.91 mmol/g in that by Gupta et al. [30,44], 136 mS/cm at 70 °C with IEC of 1.93 mmol/g in that by Wang et al. [37,38], and 102 mS/cm at 80 °C with IEC of 2.19 mmol/g in that by Mohanty et al. [20]. The DFT calculations by Castanñeda and Ribadeneira concluded that QSEBS is a stable backbone polymer to support the trimethylammonium (TMA) group at the dry polymer state [26]; however, the degradation of the headgroups is inevitable while the QSEBS is hydrated [45]. In addition, high IEC causes swelling, resulting in poor mechanical properties of the membranes.

To further improve the stability of QSEBS, the most effective way is to tether alkyl fragments, with lengths from C_3_ to C_6_, between the cationic groups and polymer backbone. Mohanty et al. [20] utilized transition metal-catalyzed C–H borylation and Suzuki coupling method to synthesize QSEBS with TMA groups alkylated by the C_6_ spacer. The morphological change was observed for SEBS–C_6_Q based on the SAXS profile. The cationic moieties enhanced hydration such that the ionic channels expanded from 31 nm to 34 nm compared with QSEBS [20]. Lin et al. also studied C_6_ alkylated QSEBS with IEC ranging from 0.51 to 1.23 mmol/g. The interdomain spacing of ionic clusters increased with IEC and WU, and their SEBS–C_6_Q with IEC of 1.23 mmol/g exhibited a maximum hydroxide conductivity of 56.4 mS/cm at 80 °C [46]. Jeon et al. developed the Friedel–Crafts Bromoalkylation method to selectively functionalize the styrene block and produced a series of alkylated QSEBS with C_3_ to C_5_ spacers at the degree of functionality (DF) of 50% to 80%. The SEBS–C_3_Q with IEC at 1.62 mmol/g had the highest conductivity of 93 mS/cm at 80 °C [32]. On the other hand, hexyl crosslinkers effectively control the swelling issue of the QSEBS with high-IEC [25,29,32]. Jeon et al. [32] combined the designs of alkyl spacers and crosslinkers and altered the degree of crosslinking for C_6_–crosslinked QSEBS. The ion conductivity for crosslinked QSEBS (IEC around 1.50 mmol/g) ranged from 29 (30 °C) to 65 (80 °C), but the WU decreased from 70 wt% to 20 wt% as the degree of crosslinking increased from 20% to 100% [32].

The above modifications of QSEBS change the polymer architecture, which controls the morphology of the hydrated polymer membrane. It has become a nontrivial task to characterize the nanostructure of the ionic domain and its efficiency in transporting hydroxide ions in specific working conditions. Molecular modeling, especially coarse-grained (CG) methods, is a convenient tool to probe the morphology of a designed product. For example, dissipative particle dynamic (DPD) simulations by Paddison and coworkers provided design guidelines for QSEBS in terms of the types of cationic groups, the style of alkyl tethering, the styrene ratio, and the hydration levels [35,37,41,47]. Molinero and coworkers focused on polyphenylene oxide (PPO) functionalized with alkylated TMA groups; detailed transport mechanisms of various polymer architectures were explored by coarse-grained molecular dynamics simulations [47,48,49]. Lee developed a scale-bridging DPD force field and modeled PPO-based and SEBS-based AEM, where membranes’ transport and structural properties based on modern synthesis ideas were examined [34,50,51]. However, even with the most efficient particle-based method with the electrostatic calculations being discarded, simulations can only tackle a system size of 42.7 nm, corresponding to the modeled polymer of molecular weight of roughly 1/8 of the commercial products [34]. To properly model the experimental QSEBS, whose characteristic morphology is usually tens of nanometers, the simulation system size should be scaled up by at least one fold. If one prefers to increase the resolution to model local chemistry [47,48,49] or preserve the long-range charge interactions for hydroxide ions [34,50,51], the spatial and temporal scales are further limited. As a result, it is crucial to develop a coarse-grained protocol that balances the accuracy and efficiency of modeling AEM. With careful model construction and force field parameterization, CG simulation can also be applied to studying other ion-conductive polymers [52] or novel materials for microbial fuel cells [50,53].

This work extends the scale-bridging DPD method [34,50,51] to studying QSEBS with alkyl side chain modification. A novel CG hydroxide model was developed here to characterize the diffusivity of ions, where the model was constructed based on the linear potential in the most popular DPD framework [51]. Distinct from the previous model based on Morse potential [33,54], the current model employs the same force types as other pairwise forces and can be easily incorporated into other DPD-based studies. This work systematically simulated a series of SEBS-C_4_Q, modified by adding alkyl side chains to explore new AEM designs with improved ion conductivities. Established on recent findings by Al Munsur et al. where SEBS-C_6_Q-C_12_ delivers conductivity twice the level of SEBS-C_4_Q at the full hydration state [25], this work probed the morphological changes for different alkyl chain lengths (C_0_ to C_24_) and hydration levels (*λ* = number of water molecules per cationic group, from 10 to 30).

## 2. Methods

### 2.1. The Chemical Structure and Coarse-Grained Model for the Designed Polymers

**Figure 1:**
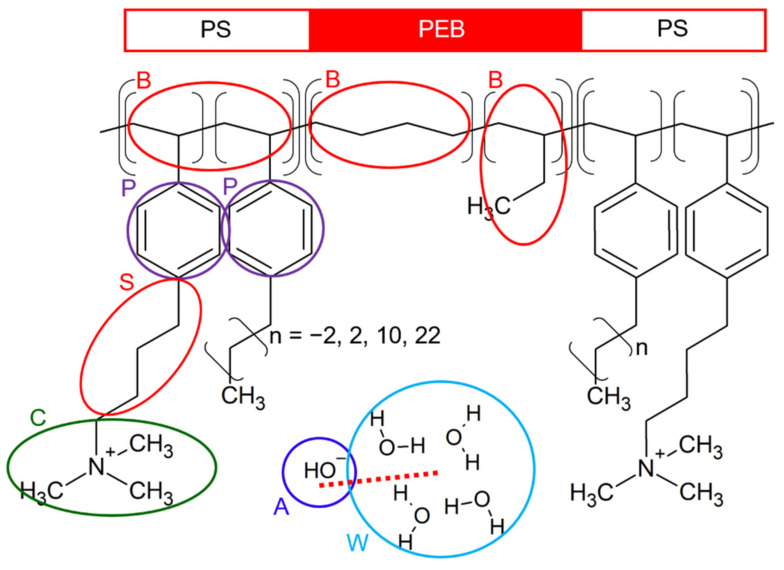
Chemical structure of the SEBS-based ionomer with pendant quaternary groups (C_4_Q) and alkyl side chains (C_0_, C_4_, C_12_, and C_24_). The coarse-graining schematic is illustrated based on the following bead type definitions: the B bead represents the alkyl chain in the PEB block, the P bead represents the phenyl ring in PS blocks, and the C bead represents the cationic TMA group. The dissociated hydroxide ion is modeled by the A bead solvated by the water bead W. The butyl fragment in both functionalized and alkylated side chains is denoted as the S bead.

Figure 1 shows the chemical structure of the butyl quaternary ammonium-functionalized alkyl chain-grafted SEBS ionomer (SEBS–C_4_Q–C_n_). The backbone of the triblock copolymer comprises polystyrene (PS) and poly-ethylene-co-butylene (PEB). The hydroxide ion-conducting head group, trimethylamine (TMA), was tethered on the PS block with a C_4_ spacer. The alkyl side chains were tethered separately, whose chain length varied from C_4_, C_12_, to C_24_. SEBS–C_4_Q–C_0_ with no additional alkyl chain was studied as a reference. Such an ionomer can be synthesized by the modified Hibb’s procedure [55] on commercial SEBS triblock copolymers [25]. The functionalized side chains were added to SEBS by Friedel-Crafts acylation. The alkyl side chains were added by further acylation with agents of different lengths (bromobutanoyl chloride for C_4_, bromododecanoyl chloride for C_12_, and etc.) Al Munsur et al. studied SEBS–C_4_Q–C_n_ (*n* = 4 and 12) and reported that SEBS–C_4_Q–C_4_ and SEBS–C_4_Q–C_12_ deliver higher ion conductivities than the non-alkylated ones and most SEBS-based AEMs in the literature. They found that the alkyl side chains create “free volume” in the polymer matrix, and the membrane absorbs more water at a controllable swelling ratio. The hydration level *λ* increased from 34 (WU of 79%) to 54 (WU of 128%) when grafting C_12_ chains to the non-alkylated SEBS–C_4_Q. The high water content sizes up the hydrophilic domain, resulting in more efficient diffusion of ions. This work advances the design by Al Munsur et al. [25], aiming to understand the influences of alkyl side chains on fixed hydration levels from a molecular perspective.

**Figure 2:**
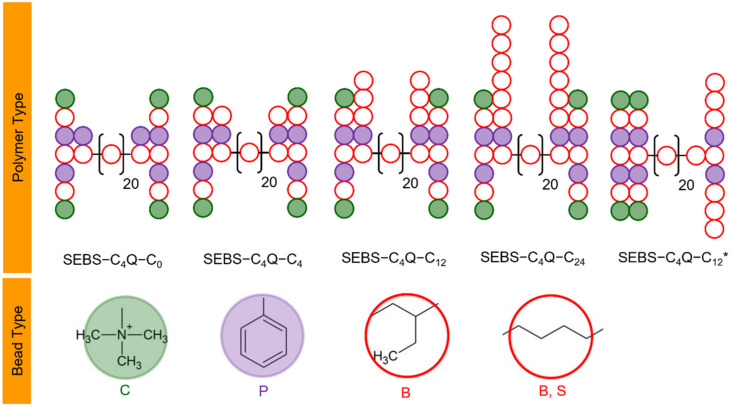
Coarse-grained models for SEBS–C_4_Q–C_n_ (*n* = 0, 4, 12, and 24) and the illustration for atom-to-bead mapping. For SEBS–C_4_Q–C_n_, alkyl side chains are tethered on both PS blocks, whereas for SEBS–C_4_Q–C_12_*, alkyl side chains are tethered on the same side.

The coarse-grained model for SEBS–C_4_Q–C_n_ was designed based on the mapping between coarse-grained beads and the corresponding groups of comparable size. As shown in Figure 1 and Figure 2, four carbon atoms mapped to a backbone (B) bead. The mapping also applies to the alkyl fragment of the side chain (S) bead. A TMA group mapped to a cationic (C) bead. As for mobile components, a hydroxide ion mapped to an anionic (A) bead, and four water molecules mapped to a water (W) bead. The mapping of the W bead to water characterizes the physical units of DPD, where the spatial and temporal units are determined based on the density and self-diffusion coefficient of water. Such coarse-graining protocols are consistent with previous DPD works [34,50,51] and the investigations by the pioneers in modeling fuel cell membranes [34,35,36,40,56,57,58]. The styrene ratio (Sty%) equals 35%, and the degree of functionality (DF) equals 67%. These compositions were set to mimic the synthesized SEBS-based ionomers with good performances [37,38]. The resulting ion exchange capacity (IEC) equaled 1.34 mmol/g to 1.72 mmol/g, where the IEC decreased with the length of the alkyl side chains. The hydration level (*λ*), denoted as the number of water molecules per cationic group, varied from 10 to 30 to cover the standard operating conditions. The water uptake (WU) can be calculated based on IEC and *λ*, ranging from 24% to 93%. The detailed system compositions are given in the Supplementary Materials.

Figure 2 illustrates the coarse-grained models for SEBS–C4Q–C_n_ ionomers based on the above mapping. The structures of SEBS–C_4_Q–C_0_, SEBS–C_4_Q–C_4_, and SEBS–C_4_Q–C_12_ differ only by the length of the alkyl side chain. In addition, SEBS–C_4_Q–C_12_* was modeled to study the effects of tethering style, where TMA functionalized side chains were tethered onto one PS block with alkyl side chains being tethered on the other. In the presence of electrostatic interactions, the computationally-expensive long-ranged Ewald summation limits the accessible modeling capacity. As a result, the molecular weight (M.W. given in the Supplementary Materials) of the polymer model was 1/40 of the commercial products (M_n_ of 10^5^ g/mol). By implicitly considering Coulombic forces, contemporary DPD studies can push the size limit further to model polymers with M.W. of 1/8 of M_n_ [34,40].

### 2.2. The Force Field Parameters for Dissipative Particle Dynamics Simulations

The simulation method combines the most popular DPD algorithms by Español, Groot, and Warren (usually regarded as the “GW method”) [51,59] and the author’s parameterization for modeling fuel cell ionomers [34,50,51]. The utilized force field and parameters are given in Section 2.2, where the construction of CG hydroxide ion is described in Section 2.3. Other global parameters are summarized in the Supplementary Materials.

A cubic box populated by DPD beads was used to model hydrated AEM, where the beads interact only through pairwise forces, as formulated in Equation (1) for a pair i and j. Dissipative force (F^D^) and random force (F^R^) account for frictional and stochastic contributions to the systems. These two forces simultaneously maintained the temperature based on the drag coefficient (*γ* = 4.5) and the corresponding noise amplitude (σ = 3 due to the fluctuation-dissipation theorem) [51]. The conservative force (F^C^) characterizes the bead–bead interactions as linear-decayed repulsion. As shown in Equation (2), the conservative force is determined by the distance between the bead centers r_ij_, bead diameter (which is also served as a short-term cutoff distance) r_c_, and repulsion parameter a_ij_. The bond forces (F^B^) in Equation (1) construct polymer molecules by the standard harmonic potential given by Equation (3). The forces acting on the nearest neighbors (referred to as 1–2 bonds) and 2nd-nearest neighbors (1–3 bonds) maintain the molecular structure of the model. All parameters for non-bonded and bonded interactions are summarized in Table 1. Except for the determination of a_WA_, which are detailed in the next section, the force field parameters were characterized based on the developed parameterization procedures [33]. To briefly summarize, the non-bonded parameters were determined in a top-down manner by linking to thermodynamic properties of bead components, such as the solubility of water in polyethylene or styrene. The bonded parameters were connected to the bottom-up molecular configurations of the homopolymer melts obtained by all-atom simulations. This scale-bridging parameterizing scheme was developed for modeling surfactants [60,61] and proton-exchange membranes [62], and it was recently applied to model AEM by Lee [34,50,51].

(1) $$F_{\mathrm{ij}}\left(r_{\mathrm{ij}}\right){=F}_{\mathrm{ij}}^{C}+F_{\mathrm{ij}}^{D}+F_{\mathrm{ij}}^{R}+F_{\mathrm{ij}}^{B}$$

(2) $$F_{\mathrm{ij}}^{C}\left(r_{\mathrm{ij}}\right)=\left\{\begin{array}{ll} a_{\mathrm{ij}}\left(1-\frac{r_{\mathrm{ij}}}{r_{c}}\right)\frac{r_{\mathrm{ij}}}{r_{\mathrm{ij}}}, & r_{\mathrm{ij}}<r_{c}\\ 0, & r_{\mathrm{ij}}\geq r_{c} \end{array}\right.$$

(3) $$U_{\mathrm{ij}}\left(r_{\mathrm{ij}}\right)=\frac{K}{2}{\left(r_{\mathrm{ij}\;}-{\;r}_{0}\right)}^{2}$$

### 2.3. The Coarse-Grained Model for Hydroxide Ions

Standard coarse-grained simulations model small ions by their hydrated state. The choice was made to have a consistent coarse-grained bead volume [34,64], but it was obscured to extract the diffusivity of ions directly from the simulation trajectories. This work presents an innovative approach to tracking the mobility of ions. As shown in Figure 1, an OH^−^ was modeled individually as an A bead, which associates with the water bead W with an attractive force, forming a quasi-particle. When an A–W quasi-particle interferes with nearby W beads, the W–A–W complex is formed, and the A bead can change its host by hopping to another W bead. The associative A–W force decelerates the A bead and controls its hopping rate based on a given *a*_WA_.

To mimic the networking of OH^−^ and nearby water molecules [2,65,66,67], a short cutoff (r_c_ = 0.6) was assigned to ensure that an A bead mainly associates with two W beads (up to 8 H_2_O). The reduced mass of the A bead was set to 0.24 (unitless), equal to the weight of a hydroxide ion divided by that of 4 water molecules. The drag coefficient *γ* for A–W pairs was tuned down to 0.5 to boost the mobility of A beads. To map the diffusivity of the A beads to the experimental diffusion coefficient of the hydroxide ion, we modeled the motion of a single A particle in the bulk W beads. Using the Einstein equation, the mean square displacement (MSD) of single-particle trajectories was used to approximate the self-diffusion coefficient of A beads and W beads (see Supplementary Materials). The experimental self-diffusion coefficients for water and the hydroxide ion at 298 K were 2.26 × 10^−9^ m^2^/s and 5.30 × 10^−9^ m^2^/s, respectively [66,68]. The strength of a_WA_ was gradually increased until the correct ratio $D_{{\mathrm{OH}}^{-}}/D_{H_{2}O}$ of 2.35 was reproduced, and the final value is reported in Table 1.

A similar model was developed [69] and applied to study proton transfer in proton-exchange fuel cell membranes [62]. At the smallest coarse-grained size, complex transport behavior such as proton hopping frequency is reproduced by DPD simulations [69]. The current model replaces the Morse potential used in previous studies [34,51,64,70] with the linear potential for A–W association, making the computational scheme more versatile. The devised “GW hydroxide model” and the parameters can be directly applied to other DPD methods for modeling the OH^−^ diffusivity at room temperature, as long as the self-repulsion parameter and drag coefficient follows the general assignment (a = 25 and *γ* = 4.5). One can also follow the protocol to recalibrate the parameter for higher temperatures. When mapping the diffusivity of W beads to that of bulk water, a scaling factor of *N* (number of H_2_O per W bead) should be applied, as suggested in the literature [70].

## 3. Results and Discussions

### 3.1. Nanostructure and Conductivity of Anion Exchange Membranes

**Figure 3:**
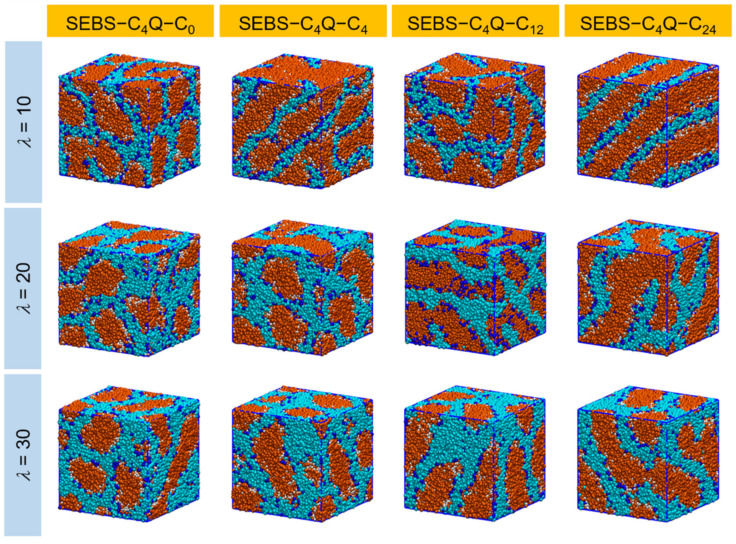
Morphologies of the nano-segregated SEBS–C_4_Q–C_x_ polymers on different hydration levels (*λ*). B/S beads are in orange, P beads in white, C beads in blue, and W and A beads in cyan. The simulation box size is roughly 30 bead diameters (21.3 nm).

Figure 3 shows the morphologies of hydrated AEM with different polymer architectures at various hydration levels. Nano-phase segregation of polymeric and water domains was observed for all designed SEBS–C_4_Q–C_n_. Generally, several main factors drive the segregation, including the miscibility between polystyrene and polyethylene blocks, the hydrophobicity of the polymeric backbone and side chains, the solvation of cationic groups, and their association with surrounding hydroxide ions. As shown in Figure 3, the cationic groups (in blue) reside on the hydrophilic and hydrophobic domain interface, forming ion-conductive pathways with water. As the water content increased, these water channels expanded and became interconnected. The shape of polymer aggregates also shifted from slab-like to spherical-like. By adding the alkyl side chains to the polymers, the hydrophobicity of the system increased, and more substantial segregation is expected. Visually speaking, the width of the water channels was seen to be affixed to the hydration level (determined by *λ*). However, the morphology evolved significantly with the alkyl chain length. At the lowest hydration *λ* = 10, the aggregates of alkylated ionomers agglomerated into less but larger clusters. Compared with the continuous water network of SEBS–C_4_Q–C_0_, the hydrophilic domain (in cyan) of alkylated ionomers was shifting to an ordered and less-connected structure. Noticeably, a well-defined lamellar structure was observed when the alkyl chain length increased to C_24_. This ordered structure was disrupted when the hydration level increased to *λ* = 20~30, where the nearby water subdomains of SEBS–C_4_Q–C_24_ bent and leaned to each other. For SEBS–C_4_Q–C_4_ and SEBS–C_4_Q–C_12_, the coalescing of the water channels formed nodes at the highest water content *λ* = 30. This local aggregation of water expanded the maximum ion conductive pathway. Nevertheless, it could also cause the formation of the bottleneck due to excessive self-assembly behavior, as discussed in earlier studies [71].

**Figure 4:**
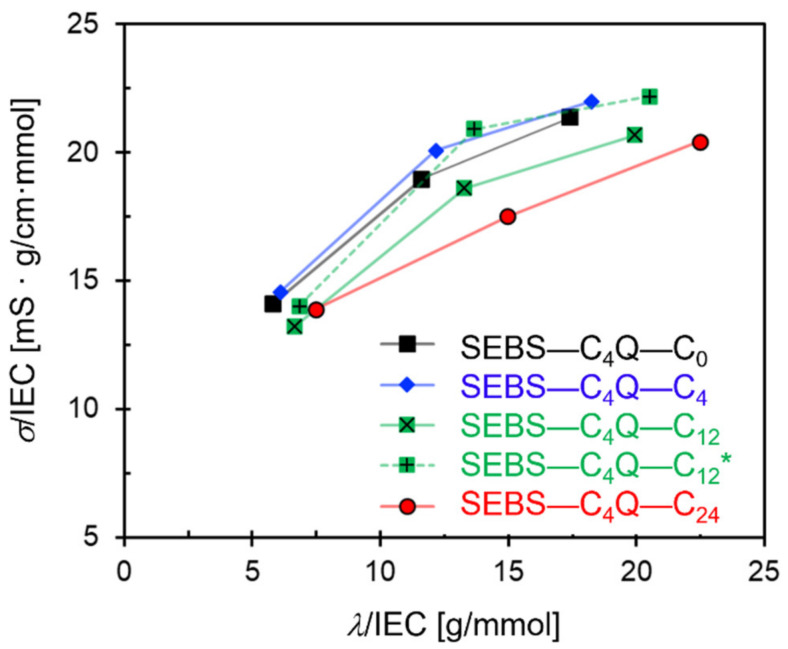
Conductivity (*σ*) versus hydration level (*λ*) standardized by IEC for five ionomer designs. The polymer types are given in Figure 2.

Based on the calculated MSD of the hydroxide beads, ion conductivity was estimated by using the Nernst–Einstein equation (see Supplementary Materials for details). As expected, the conductivities grew monotonically with the hydration level, as shown in Figure 4. The high water content helped in developing the water network, and the expanded water channels improved the diffusivity of the mobile components. Each designed ionomer had a different growth rate in the conductivity against hydration level, which is related to the interplay of the local interactions, as discussed in the following subsection. The conductivity slightly decreased with the length of the alkyl side chain at the same hydration level, which is consistent with the decrease in IEC and WU due to the changes in polymer structures. The hydrophobicity provided by alkyl side chains maintained the nano-segregation, which delivered a similar ion conductivity at a lower IEC than the non-alkylated ones. As shown in Figure 4, the conductivities normalized by IEC were around 15 (mS·g/cm·mmol) at the lowest hydration level, and the values increased to approximately 20 at the highest hydration level. Table 2 summarizes the transport and structural properties of all SEBS–C_4_Q–C_n_ ionomers with alkyl side chains of C_0_ (no alkyl side chain), C_4_, C_12_, and C_24._

### 3.2. The Effects of the Alkyl Side Chains

**Figure 5:**
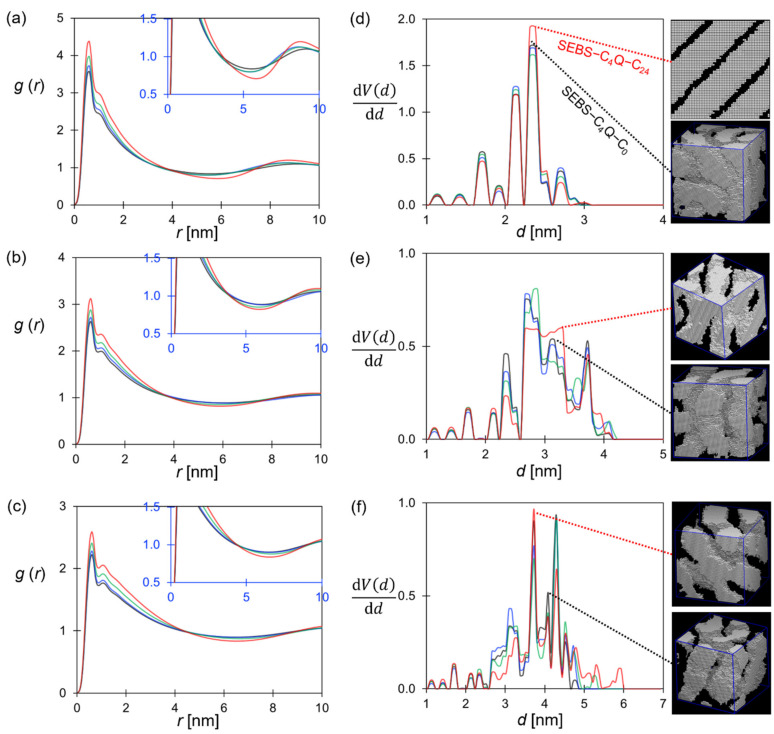
Structural analysis of ionic pathways: RDF of W–W at (**a**) *λ* = 10, (**b**) 20 and (**c**) 30. PSD of hydrophilic domains at (**d**) *λ* = 10, (**e**) 20, and (**f**) 30. Line color: SEBS–C_4_Q–C_0_ (black), SEBS–C_4_Q–C_4_ (blue), SEBS–C_4_Q–C_12_ (green), SEBS–C_4_Q–C_24_ (red).

The radial distribution function (RDF) of the W beads is the straightforward measure that characterizes the size and correlation of the hydrophilic domain. The position where the g(r_WW_) starts to decorrelate, i.e., passing through unity after the 1st peak, gives a rough estimate for the size of water clusters. As shown in Figure 5a–c, the cluster size was around 3 nm to 4 nm. The 1st and the 2nd peaks were higher for alkylated SEBS–C_4_Q–C_n_ ionomers, suggesting the water network was more structured. Specifically, the prominent 2nd peak was observed for the lamellar structure formed by SEBS–C_4_Q–C_24_. The hydrophilic domain (width of the first peak) also grew slightly with increased alkyl chain length, showing that the excess hydrophobicity of alkyl chains enhanced the aggregation of water. The observations qualitatively agree with the AFM phase images of Al Munsur et al. [25], where the correlation of nearby water clusters was increased by introducing alkyl side chains. It was argued that the free volume [72,73,74] formed by the self-assembled SEBS ionomers increases the equilibrium WU substantially with the alkyl chain length. The hydration level was 54 (WU = 127 wt%) for a SEBS–C_4_Q–C_12_ with IEC equal to 1.5 mmol/g, and the excess water contributed to the percolation of the hydrophilic domain [25]. The dependence of hydrophilic domain size on the hydration level was qualitatively reproduced in the simulation. The distance of decorrelation increased with the hydration level, suggesting the formation of an exclusive water domain.

The pore size distribution (PSD) of the equilibrium membrane structure was analyzed to characterize the structural details of the ionic pathways. As shown in the embedded visualization in Figure 5d–f, the simulation systems were first digitized into lattice models, where lattice sites overlapped with W beads were regarded as pores. The porous region represents the percolated hydrophilic domain, where the geometry was probed by a hard-sphere particle using PoreBlazer software [75]. The pore size distribution is illustrated in Figure 5d–f, and the maximum developed pore width (d_max_) and the bottleneck (d_min_) are reported in Table 2. For all SEBS–C_4_Q–C_n_ ionomers, the characteristic channel width was between 2 to 3 nm at *λ* = 10, and the SEBS–C_4_Q–C_24_ had a more uniform channel width centered at about 2.5 nm, as shown in Figure 5d. As the hydration level increased, the whole distribution shifted to the larger values, resulting in more developed water channels. As indicated in Table 2, as *λ* increased from 10 to 30, the growths in the bottleneck and the maximum channel widths of SEBS–C_4_Q–C_0_ were 1.6 nm and 2 nm, respectively. For SEBS–C_4_Q–C_4_ and SEBS–C_4_Q–C_12_, the dependence of the channel geometry on the hydration level was similar. Despite the substantial increase in the channel widths, the smallest probed pores did not vanish as *λ* increased, as shown in the peaks of PSD around 1 to 2 nm. At higher hydration levels, nearby water clusters agglomerated into more prominent nodes. The unevenly distributed aqueous domain could cause the formation of more bottlenecks in the water network and affect the transport properties of mobile components. The situation was especially noticeable for SEBS–C_4_Q–C_24_ at *λ* = 30. As shown in Figure 5f, the clusters of 5~6 nm were developed, but the maximum channel geometries increased by less than 1 nm compared with those at *λ* = 10.

Although the effects of alkyl chain length were qualitatively reproduced in simulations, it is noted that the obtained water channel size was much smaller than the values estimated from the SAXS diagram [25]. The ionic peaks were around 0.029 Å^−^^1^ (SEBS–C_4_Q–C_0_) to 0.022 Å^−^^1^ (SEBS–C_4_Q–C_12_), corresponding to the water domain sized at 22 nm to 29 nm [25]. As discussed above, the much smaller characteristic length scale of the nanostructure is due to the shorter polymer model constructed. Nevertheless, the explored morphological changes for different polymer architectures provide helpful insights, as also argued in other DPD studies on commercial SEBS [36]. In summary, membranes with C_4_ alkyl chains develop the water network with better transport efficiency, as quantified by D_W_/D_W_bulk_ reported in Table 2. It was first seen that SEBS–C_4_Q–C_4_ delivered a higher ion conductivity with a lower IEC and WU than SEBS–C_4_Q–C_0_. SEBS–C_4_Q–C_12_ had a similar channel structure as SEBS–C_4_Q–C_4_, and hence the growth rate of ionic conductivity against hydration level was also alike, as shown in Figure 4.

**Figure 6:**
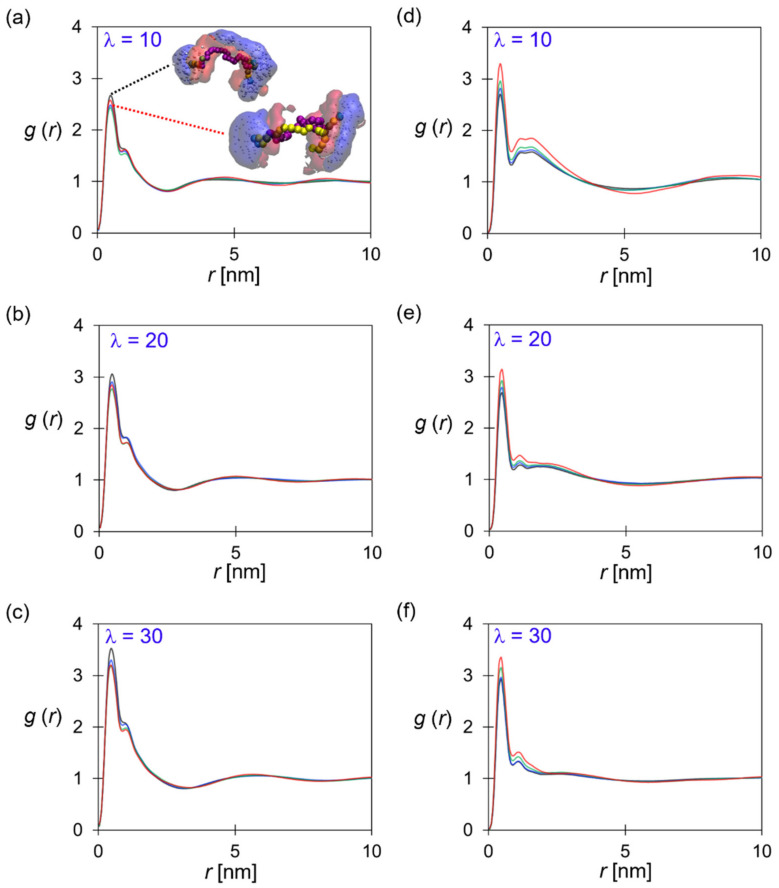
Structural analysis of polymeric domain: RDF of P–P at (**a**) *λ* = 10, (**b**) 20, and (**c**) 30. RDF of C–C at (**d**) *λ* = 10, (**e**) 20, and (**f**) 30. Line color: SEBS–C_4_Q–C_0_ (black), SEBS–C_4_Q–C_4_ (blue), SEBS–C_4_Q–C_12_ (green), SEBS–C_4_Q–C_24_ (red).

The polymeric domain partially controls the structure of ionic pathways. Figure 6a–c show the distribution of the P (styrene) beads in terms of the *g*(*r*_PP_), which is related to the conformation of SEBS–C_4_Q–C_n_ in the nano-segregated AEM. As illustrated in Figure 6a, the PEB blocks (pink beads) packed into the hydrophobic core. The tethered alkyl side chains (yellow beads) aligned with the PEB block, altering the size and shape of the hydrophobic domain. The pendant TMA reached out to the anion-rich, hydrophilic domain (blue cloud), and the P beads (red cloud) were distributed between the hydrophilic and hydrophobic domains.

In general, longer alkyl chains resulted in the lower 1st peak along with the broader 2nd peak in the *g*(*r*_PP_), suggesting the development of the larger hydrophobic domain and more distributed styrene blocks. On the other hand, increasing the hydration level made the 1st peak of *g*(*r*_PP_) more pronounced, as shown in Figure 6a–c. In Figure 3, the excess water at *λ* = 30 caused SEBS–C_4_Q–C_0_ polymers to form clusters with spherical-like cross-section, and the styrene fragments are more concentrated in this morphology. While the alkyl side chain and water content have opposite effects on the PS block distribution, it is possible to engineer a specific membrane structure at a given condition by altering the alkyl chain length.

In Figure 6d–f, the distributions of cationic groups are compared. It was seen that the intensity of the 1st peak in *g*(*r*_CC_) was less sensitive to the hydration level. However, a prominent 2nd peak was observed at *λ* = 10, which corresponds to the correlation of TMA groups on the opposite sides of the water channels. While the hydration level increased, the 2nd peak weakened due to the expansion of the water channel. Longer alkyl chains lead to more well-defined hydrophilic–hydrophobic interfaces, thus affecting the distribution of TMA groups on the structured morphology. With the increasing alkyl chain length, the 1st peak in *g*(*r*_CC_) was intensified due to the enhanced phase separation, and the elevation of the 2nd and 3rd peaks indicated the development of the ordered structure.

**Figure 7:**
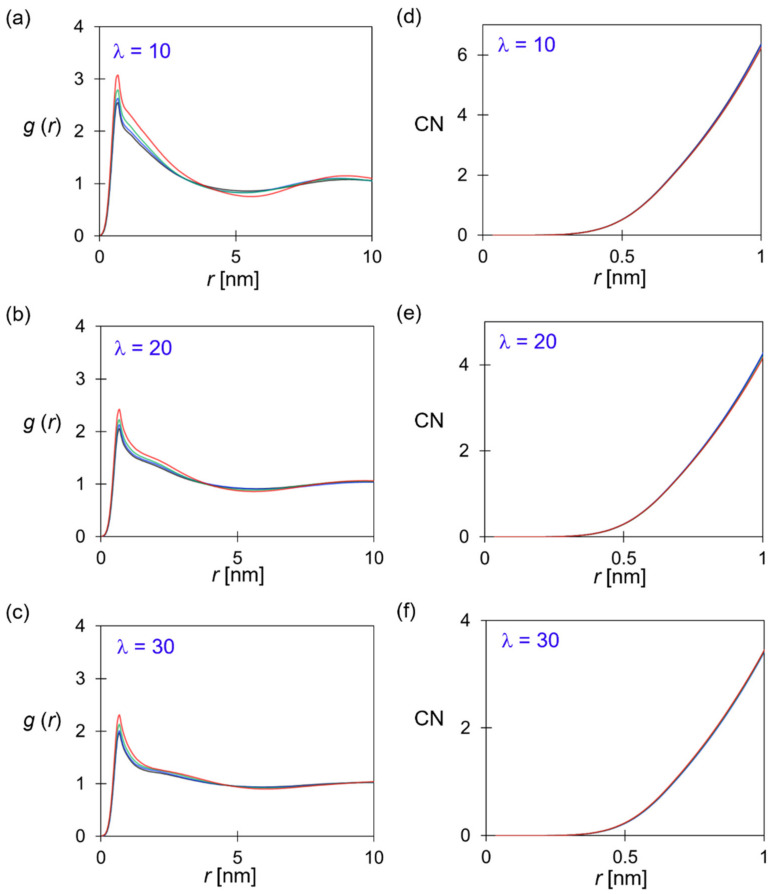
Association between hydroxide and cationic groups: RDF of C–A at (**a**) *λ* = 10, (**b**) 20, and (**c**) 30. The coordination number (CN) of A against C at (**d**) *λ* = 10, (**e**) 20, and (**f**) 30. Line color: SEBS–C_4_Q–C_0_ (black), SEBS–C_4_Q–C_4_ (blue), SEBS–C_4_Q–C_12_ (green), SEBS–C_4_Q–C_24_ (red).

Figure 7 shows the association of the cationic group and hydroxide anions in terms of *g*(*r*_CA_) and the running coordination number of OH^−^ near a TMA group. As anticipated, the higher water content broadened the water domain, and more OH^−^ ions were associated with the bulk water when diffusing through the membrane, as evidenced by the descending 1st peak in Figure 7a–c. The flattened correlation at larger length scales implies a fully-developed water network. Similar to the discussion of *g*(*r*_CC_), the more apparent nano-phase segregation led to the stronger association of ion pairs, which could play a role in stabilizing the hydrated morphologies via the Coulombic attraction. Although the C–A pairs showed stronger association at longer alkyl chains, the lower IEC and the more dilute charged beads (given in Table 2) concurrently made similar coordination number (CN) profiles for all ionomers. At the distance corresponding to the bead diameter (~0.7 nm), the CN was around two at *λ* = 10, and the value decreased to 1 at *λ* = 30. This result is consistent with the atomistic simulations [28,76,77], where the transport of the hydroxide ions was mainly through vehicular mechanisms at the similar water content in this study. As reported in Table 2, systems with more uniform channel width and the TMA distributions featured higher values of D_A_/D_W_.

### 3.3. The Effects of the Side-Chain Tethering Style

**Figure 8:**
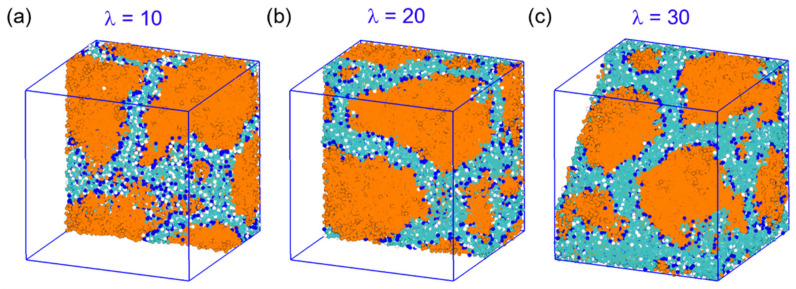
Visualization of SEBS–C_4_Q–C_12_* at the hydration level of (**a**) *λ* = 10, (**b**) *λ* = 20, and (**c**) *λ* = 30. The materials are clipped for presenting ionic pathways. The colors of the beads are the same as in Figure 3.

**Figure 9:**
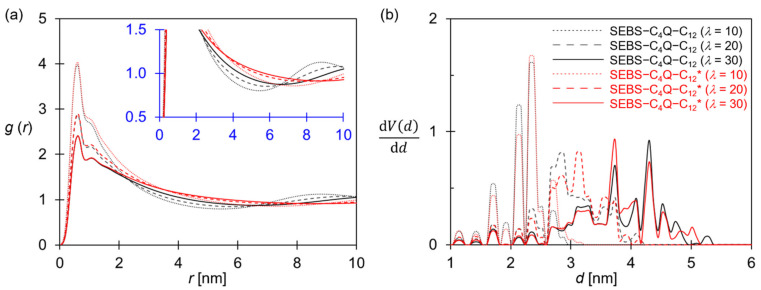
(**a**) RDF of W–W and (**b**) PSD of SEBS–C_4_Q–C_12_ (black lines) and SEBS–C_4_Q–C_12_* (red lines) at the hydration level of 10 (dotted lines), 20 (dashed lines), and 30 (solid lines). The polymer types are given in Figure 2.

Two ionomers, SEBS–C_4_Q–C_12_ and SEBS–C_4_Q–C_12_*, were modeled to explore the effects of the side-chain tethering style. As illustrated in Figure 2, SEBS–C_4_Q–C_12_ had the alkyl side chains tethered on both PS blocks, but SEBS–C_4_Q–C_12_* had the alkyl side chains tethered on the same PS block of one side. In Figure 8, the morphologies of the hydrated SEBS–C_4_Q–C_12_* are compared. Similar to SEBS–C_4_Q–C_12_, nano-phase segregation was observed with developed water networks. The similar structural profiles for the two designs in Figure 9 indicate the membrane morphology is mainly controlled by IEC and water content. However, the W–W distribution functions of SEBS–C_4_Q–C_12_* in Figure 9a decay slower and broader at all hydration levels, suggesting that a larger continuous water domain developed. At *λ* = 10 and 20, the peaks of PSD for SEBS–C_4_Q–C_12_* systematically shifted to the larger values, corresponding to the larger channel width as given in Table 2. At *λ* = 30, the PSD profile of SEBS–C_4_Q–C_12_* was slightly more uniform than that of SEBS–C_4_Q–C_12_. The peak with the highest intensity was at 3.7 nm compared to 4.2 nm for SEBS–C_4_Q–C_12_. These findings also suggest that more uniformly distributed water networks developed in the SEBS–C4Q–C12* system, as shown in Figure 8.

**Figure 10:**
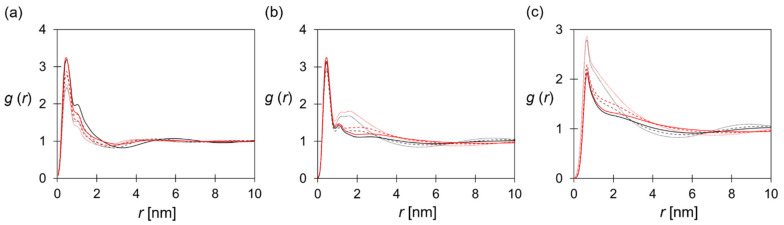
RDF of (**a**) P–P, (**b**) C–C, and (**c**) C–A for SEBS–C_4_Q–C_12_ (black lines) and SEBS–C_4_Q–C_12_* (red lines) at the hydration level of 10 (dotted lines), 20 (dashed lines) and 30 (solid lines).

Compared with SEBS–C_4_Q–C_12_, the polymer subdomains of SEBS–C_4_Q–C_12_* were more uniform. As shown in Figure 8, the ionomers formed spherical aggregates at *λ* = 30. Tethering functionalized side chains on one polymer end-block made SEBS–C_4_Q–C_12_* similar to ionic surfactants, and the aggregation of polymer molecules was analogous to surfactants self-assembling into spherical micelles. In addition, the concentrated functionalized side chains led to excess solvation for their tethered block. The styrene beads (white beads) were present in the hydrophilic domain (Figure 8), which corresponds to the lower 2nd peak and flattened *g*(*r*_PP_) profile in Figure 10a. Figure 10b shows the correlation of cationic beads. Both ionomers feature the phenomenal 2nd peak in *g*(*r*_CC_) as observed in Figure 6b at *λ* = 10, and the peak decreased as the hydration level increased. The hydrated SEBS–C_4_Q–C_12_* formed more uniform water channels than SEBS–C_4_Q–C_12_, where the aqueous domain is evenly distributed, corresponding to the higher 2nd peak of *g*(*r*_CC_) in Figure 10b. The uniform water network and the distributed cationic groups made the TMA–OH^−^ association more substantial, as shown in Figure 10c. The lower D_A_/D_W_ for SEBS–C_4_Q–C_12_* given in Table 2 suggests the average channel width was slightly smaller, so the electrostatic attractions from the cationic groups decreased the hydroxide ion diffusivity. Nevertheless, the higher D_W_/D_W_bulk_ indicated that high-efficiency ion-conductive pathways were formed, resulting in higher ion conductivities.

## 4. Conclusions

Mesoscale method DPD simulation was used to investigate the morphology and the transport properties of a series of butyl quaternary ammonium-functionalized SEBS with alkyl side chains, SEBS–C_4_Q–C_n_, with different alkyl chain lengths (*n* = 0, 4, 12, and 24) and hydration levels (*λ* = 10, 20, and 30). For the reference SEBS–C_4_Q–C_0_ system, the interconnected water network was formed by nano-segregation. The hydrophilic domain was developed with high water content, featuring larger water channels and more efficient ion transport. By tethering the alkyl side chains, membrane morphology evolved significantly due to the hydrophobicity of side chains, even with the shortest C_4_ side chain. The interconnected structure shifted to the lamellar one for SEBS–C_4_Q–C_24_ at *λ* = 10. Overall, the alkylated SEBS–C_4_Q–C_n_ delivered similar ion conductivity with lower ion exchange capacity than the non-alkylated one.

Detailed analysis suggests that the correlation length of the water domain increased with the alkyl chain length. The hydrophilic phase also transformed into a more ordered structure, as shown in the RDF of W beads and PSD profiles. The alkyl side chains aligned with the PEB backbone and assembled into an extensive polymeric phase. The broad distribution of P beads indicates the development of the hydrophobic domain. The ordered nanostructure of alkylated ionomers strengthens the association of cationic groups and hydroxide ions, which impacts the transport efficiency of ions. SEBS–C_4_Q–C_4_ delivered higher ion conductivity than SEBS–C_4_Q–C_0_ at a lower IEC and WU. Tethering longer alkyl chains altered the morphology of hydrated ionomers, but the water domains could be unevenly distributed. A uniformly developed water network was bound for high conductivity, and the uniformly sized polymeric aggregates were hooked to good mechanical strength.

By tethering all alkyl side chains to one side of the triblock copolymer, the nano-segregation shifted to a more ordered structure for SEBS–C_4_Q–C_12_*. The slow decay of RDF of W implies the percolation of the water domain, and the PSD and diffusivity of W beads indicate the formation of a well-connected water network. The ion conductivities were systematically improved for SEBS–C_4_Q–C_12_* than SEBS–C_4_Q–C_12_. The findings are controversial to another investigation on PPO-based ionomers, where the transport properties were insensitive to polymer architectures [48]. It could have resulted from the higher flexibility and the hydrophobicity of the aliphatic backbone, and the morphological changes corresponding to polymer structure differ from the aromatic backbone.

The utilized DPD and the hydroxide model presented in this work are versatile tools to characterize synthesized anion-conductive polymers. Alkylation is an effective way to control the morphology of the quaternized SEBS membranes. Developing a more efficient coarse-grained protocol is important to quantitatively model the microstructure of the synthesized SEBS-based polymers, where the characteristic length of water clusters and spacing are often more than 20 nm.

## Figures and Tables

**Table 1:** Parameters for non-bonded and bonded interactions.

Repulsion Parameters					
a_ij_	B/S ^a^	C	P	W	A
B/S	25.0				
C	46.5	25.0			
P	30.4	41.2	25.0		
W	46.5	41.2	46.5	25.0	
A	25.0	25.0	25.0	−8.3	25.0
**Bond Parameters**					
Backbone ^b^	K_(12)_	r_0(12)_	K_(13)_	r_0(13)_	
	240.0	0.6	12.0	1.7	
Side chain ^c^	K_(12)_	r_0(12)_	K_(13)_	r_0(13)_	
	80.0	0.8	40.0	1.6	

^a^: Beads type B and S are considered the same due to similar bead components (as shown in Figure 2). ^b^: K is the bond stiffness and *r*_0_ is the equilibrium bond length. The subscripts (12) and (13) refer to 1–2 bonds and 1–3 bonds. ^c^: Parameters for side chains were obtained based on Vishnyakov et al. [63].

**Table 2:** Transport and structural properties of SEBS–C_4_Q–C_n_.

Polymer	IEC [mmol/g]	*λ* ^a^	WU ^b^ (wt%)	D_W_/D_W_bulk_^c^	D_A_/D_W_ ^c^	*σ* ^d^ [mS/cm]	d_min_ ^e^ (nm)	Err.	d_max_ ^e^ (nm)	Err. ^f^
SEBS–C_4_Q–C_0_	1.72	10	31%	0.48	1.96	24.3	2.0	0.1	3.0	0.1
SEBS–C_4_Q–C_0_	1.72	20	62%	0.65	2.31	32.7	2.8	0.1	4.1	0.1
SEBS–C_4_Q–C_0_	1.72	30	93%	0.69	2.88	36.8	3.6	0.1	5.0	0.2
SEBS–C_4_Q–C_4_	1.64	10	30%	0.52	1.85	24.0	2.1	0.1	3.0	0.1
SEBS–C_4_Q–C_4_	1.64	20	59%	0.58	2.74	33.0	2.9	0.0	4.1	0.1
SEBS–C_4_Q–C_4_	1.64	30	89%	0.69	2.92	36.2	3.5	0.2	5.1	0.4
SEBS–C_4_Q–C_12_	1.51	10	27%	0.44	1.94	19.9	2.1	0.1	2.9	0.0
SEBS–C_4_Q–C_12_	1.51	20	54%	0.58	2.44	28.0	2.8	0.2	4.0	0.1
SEBS–C_4_Q–C_12_	1.51	30	81%	0.65	2.79	31.1	3.6	0.1	5.1	0.2
SEBS–C_4_Q–C_24_	1.34	10	24%	0.47	1.82	18.6	2.1	0.1	3.0	0.1
SEBS–C_4_Q–C_24_	1.34	20	48%	0.51	2.50	23.4	2.3	0.3	3.3	0.6
SEBS–C_4_Q–C_24_	1.34	30	72%	0.63	2.69	27.3	2.5	0.6	3.7	1.1
SEBS–C_4_Q–C_12_*	1.46	10	26%	0.49	1.74	20.5	2.2	0.1	3.1	0.1
SEBS–C_4_Q–C_12_*	1.46	20	53%	0.60	2.53	30.6	2.9	0.0	4.1	0.1
SEBS–C_4_Q–C_12_*	1.46	30	79%	0.67	2.77	32.5	3.4	0.1	5.2	0.4

^a^: hydration level. ^b^: water uptake (wt%). ^c^: D_W_ and D_A_/D_W__bulk: diffusion coefficient of water bead and hydroxide bead compared to that of bulk water. ^d^: ion conductivity. ^e^: limiting and maximum diameters of the developed pore. ^f^: the standard deviation. The definition of IEC, *λ*, and WU can be found in the Supporting Information of previous work [33].

## Data Availability

The data supporting the findings of this study are available from the corresponding author upon reasonable request.

## Supplementary Materials

The following Supplementary Materials can be downloaded at: https://www.mdpi.com/article/10.3390/polym14142860/s1, Table S1: Detailed composition of the simulation systems; Figure S1: MSD of bead A and bead W versus reduced time for tracking the single-particle displacement. References [33,51,70,75,76,77,78,79,80,81,82] are cited in the supplementary materials.
