# Supplementary material for: Anion Exchange Membranes for Fuel Cells Based on Quaternized Polystyrene-b-poly(ethylene-co-butylene)-b-polystyrene Triblock Copolymers with Spacer-Sidechain Design
Source: Polymers (Basel). 2022 Jul 13;14(14):2860. doi: 10.3390/polym14142860 (PMC9317406; doi:10.3390/polym14142860)
Supplement: Supplementary file 1 [file polymers-14-02860-s001.zip › polymers-1813267-supplementary.pdf]

Supplementary Materials

Anion Exchange Membranes for Fuel Cells Based on Quaternized Polystyrene-*b*-poly(ethylene-*co*-butylene)-*b*-polystyrene Triblock Copolymers with Spacer-Sidechain Design

Qun-Gao Chen [1] and Ming-Tsung Lee [1,*]

1 Department of Chemical Engineering and Biotechnology, National Taipei University of Technology, Taipei 10608, Taiwan
* Correspondence: mtlee@ntut.edu.tw

S1. The Composition of Simulation Systems

Table S1 provides the detailed composition of the systems studied in the manuscript. The numbers of B, S, P, and C beads are given per polymer molecule for clarity.

Table S1. Detailed composition of the simulation systems

Polymer	λ	$N_{polymer}$	N_W	N_A	N_B/poly	N_S/poly	N_P/poly	N_C/poly	M.W.
SEBS–C_4Q–C_0	10	1687	16870	6748	24	4	6	4	2320
SEBS–C_4Q–C_0	20	1396	27920	5584	24	4	6	4	2320
SEBS–C_4Q–C_0	30	1191	35730	4764	24	4	6	4	2320
SEBS–C_4Q–C_4	10	1620	16200	6480	24	6	6	4	2432
SEBS–C_4Q–C_4	20	1350	27000	5400	24	6	6	4	2432
SEBS–C_4Q–C_4	30	1157	34710	4628	24	6	6	4	2432
SEBS–C_4Q–C_{12}	10	1500	15000	6000	24	10	6	4	2657
SEBS–C_4Q–C_{12}	20	1265	25300	5060	24	10	6	4	2657
SEBS–C_4Q–C_{12}	30	1094	32820	4376	24	10	6	4	2657
SEBS–C_4Q–C_{24}	10	1350	13500	5400	24	16	6	4	2994
SEBS–C_4Q–C_{24}	20	1157	23140	4628	24	16	6	4	2994
SEBS–C_4Q–C_{24}	30	1012	30360	4048	24	16	6	4	2994
SEBS–C_4Q–C_{12}^*	10	1500	15000	6000	24	10	6	4	2657

SEBS–C_4Q–C_{12}*	20	1265	25300	5060	24	10	6	4	2657
SEBS–C_4Q–C_{12}*	30	1094	32820	4376	24	10	6	4	2657

S2. Parameterization for GW Hydroxide Model

To obtain the associative potential of the GW hydroxide model described in the manuscript, we model a single A bead in a 10 × 10 × 10 box filled with 3000 W beads to parameterize the A-W association. After 10000-steps run for equilibrating the system, the simulation runs for 1 million steps, where the trajectories are recorded every 10 steps (timestep 0.01 *t*). The MSD of the A bead and a randomly selected W bead is computed by tracking the displacement of the particle. As shown in Fig. S1, the profile of MSD for W beads and A beads are in the linear region. We truncate the first 20% tracking time and estimate the diffusion coefficient based on the slope of the lines. We repeated ten simulations for each a_{WA} value, and the absolute relative error is about 0.1.

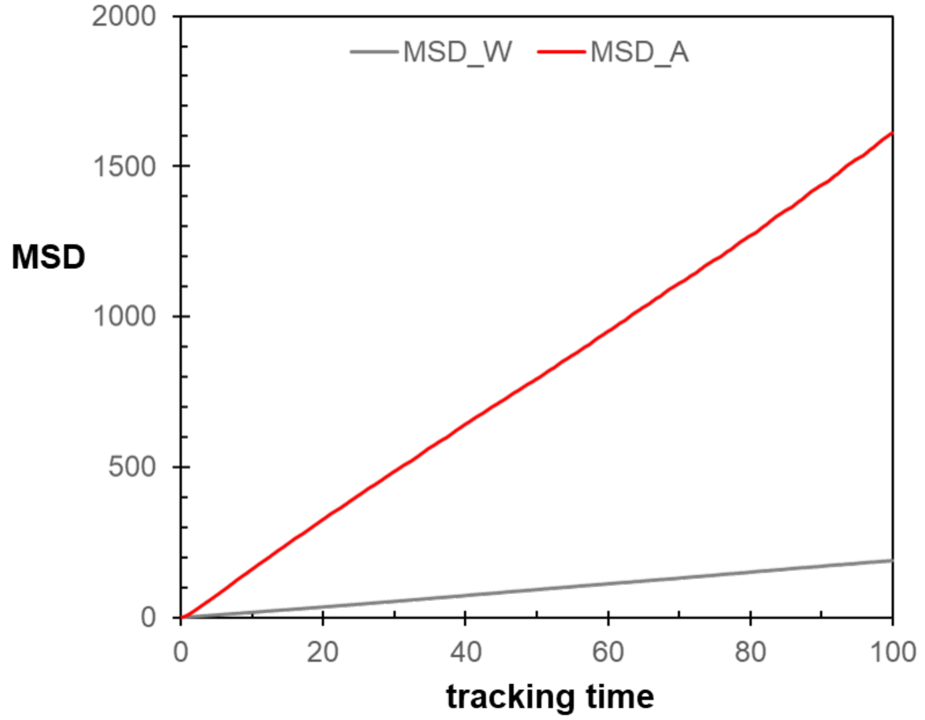

Figure S1. MSD of bead A and bead W versus reduced time for tracking the single-particle displacement.

S3. The Details for Simulations and Analysis

The DL_MESO[1] mesoscale simulation package (version 2.7 revision 9) is used to generate the initial configurations and perform DPD simulations. Systems are constructed at the density of 3 (beads/r_c^3) in a periodic unit cell whose side length equals 30 bead diameters (21.3 nm). The physical unit of r_c of 7.1 Å is obtained by mapping the density of W bead to that of water. Simulation runs for 2 million steps with 0.01 τ timestep at the isothermal-isobaric ensemble, where the reduced pressure and temperature are set to 23.7 (1 atm) and 1.0 (300 K), respectively. The friction coefficient and the relaxation time for the NPT ensemble are set to 0.5 and 10[2]. The DPD time unit τ equals 281.9 ps, as obtained by mapping the MSD of bulk W beads to the experimental diffusion coefficient of water at the room temperature[3].

The point charges on C (cationic) and A (anionic) beads are smeared by the Slater-type function[4] with the smeared length of 0.25 to avoid divergence in the calculation of Coulombic forces. The long-range electrostatic interactions are solved by the smooth particle-mesh Ewald (SPME) algorithm. The runtime parameters follow the literature in modeling ionic surfactants[5], with the *k*-vectors being increased to the current box size to ensure that the

relative error in calculating the electrostatic potential is below 1%. The permittivity equals 12.6, which scales with the coarse-grained size[6].

VMD (version 1.9.4a51)[7] is used to visualize the simulation trajectories and calculate the radial distribution function. PoreBlazer (version 4.0)[8] is used to analyze the geometry of water channels. The system is first dissected into a lattice model, where a lattice site is considered a pore if any W bead falls within one-r_c distance. The porous regions represent the hydrophilic domain, whose geometry is sampled by a hard-sphere probe. Tranal utilities of MDynaMix (version 5.2.8)[9] were used to compute the MSD of W and A beads. One thousand W and A beads are chosen separately to compute the diffusivity of water and hydroxide ions. For the last 1.5 million simulation steps, 3000 configurations are evenly sampled for MSD calculations, 300 frames for RDF analysis, and four frames for PSD analysis.

The diffusion coefficients reported in Table 2 are determined based on the standard Einstein relation using MSD−time plots, where $D_A = \langle [R_i(t_0 + t) - R_i(t_0)]^2 \rangle / 6t$ with the angle brackets being the ensemble average. The ion conductivities are estimated by the Nernst-Einstein equation, $\sigma = D_A c_A F^2 / RT$, where c_A is the concentration of the A beads (hydroxide ions), and F is the Faraday constant (F^2/RT = 3.7554×10^6 s·S/mol at room temperature). D_A is the diffusivity of the hydroxide ions, whose physical unit is obtained by comparing the MSD with the bulk W beads (D_{W_bulk}). Although the Nernst-Einstein approximation is strictly valid for the dilute ion condition, it has been used to qualitatively estimate the conductivity of fuel cell membranes in simulations[10,11].

References

1. Groot, R.D.; Warren, P.B. Dissipative particle dynamics: Bridging the gap between atomistic and mesoscopic simulation. *Journal of Chemical Physics* **1997**, *107*, 4423-4435, doi:10.1063/1.474784.
2. Lee, M.T. Designing Highly Conductive Block Copolymer-Based Anion Exchange Membranes by Mesoscale Simulations. *J. Phys. Chem. B* **2021**, *125*, 2729-2740, doi:10.1021/acs.jpcb.0c10909.
3. Easteal, A.J.; Price, W.E.; Woolf, L.A. DIAPHRAGM CELL FOR HIGH-TEMPERATURE DIFFUSION MEASUREMENTS - TRACER DIFFUSION-COEFFICIENTS FOR WATER TO 363-K. *Journal of the Chemical Society-Faraday Transactions I* **1989**, *85*, 1091-1097, doi:10.1039/f19898501091.
4. Gonzalez-Melchor, M.; Mayoral, E.; Velazquez, M.E.; Alejandre, J. Electrostatic interactions in dissipative particle dynamics using the Ewald sums. *Journal of Chemical Physics* **2006**, *125*, doi:10.1063/1.2400223.
5. Anderson, R.L.; Bray, D.J.; Del Regno, A.; Seaton, M.A.; Ferrante, A.S.; Warren, P.B. Micelle Formation in Alkyl Sulfate Surfactants Using Dissipative Particle Dynamics. *Journal of Chemical Theory and Computation* **2018**, *14*, 2633-2643, doi:10.1021/acs.jctc.8b00075.
6. Groot, R.D.; Rabone, K.L. Mesoscopic simulation of cell membrane damage, morphology change and rupture by nonionic surfactants. *Biophysical Journal* **2001**, *81*, 725-736, doi:10.1016/s0006-3495(01)75737-2.
7. Humphrey, W., Dalke, A. and Schulten, K. VMD - Visual Molecular Dynamics. *J. Molec. Graphics* **1996**, *14*, 33-38.
8. Sarkisov, L.; Bueno-Perez, R.; Sutharson, M.; Fairen-jimenez, D. Material Informatics with PoreBlazer v4.0 and CSD MOF Database. *ChemRxiv.* **Sep 6, 2020**, Preprint, doi:10.26434/chemrxiv.12923558.v1.
9. Lyubartsev, A.P.; Laaksonen, A. M.DynaMix - a scalable portable parallel MD simulation package for arbitrary molecular mixtures. *Computer Physics Communications* **2000**, *128*, 565-589, doi:10.1016/s0010-4655(99)00529-9.
10. Kreuer, K.D.; Paddison, S.J.; Spohr, E.; Schuster, M. Transport in proton conductors for fuel-cell applications: Simulations, elementary reactions, and phenomenology. *Chemical Reviews* **2004**, *104*, 4637-4678, doi:10.1021/cr020715f.
11. Kusoglu, A.; Weber, A.Z. New Insights into Perfluorinated Sulfonic-Acid lonomers. *Chemical Reviews* **2017**, *117*, 987-1104, doi:10.1021/acs.chemrev.6b00159.
